# Plasmon-induced charge separation: chemistry and wide applications

**DOI:** 10.1039/c7sc00031f

**Published:** 2017-02-10

**Authors:** Tetsu Tatsuma, Hiroyasu Nishi, Takuya Ishida

**Affiliations:** a Institute of Industrial Science , The University of Tokyo , 4-6-1 Komaba, Meguro-ku , Tokyo 153-8505 , Japan . Email: tatsuma@iis.u-tokyo.ac.jp

## Abstract

Recent development of nanoplasmonics has stimulated chemists to utilize plasmonic nanomaterials for efficient and distinctive photochemical applications, and physicists to boldly go inside the “wet” chemistry world. The discovery of plasmon-induced charge separation (PICS) has even accelerated these trends. On the other hand, some confusion is found in discussions about PICS. In this perspective, we focus on differences between PICS and some other phenomena such as co-catalysis effect and plasmonic nanoantenna effect. In addition, materials and nanostructures suitable for PICS are shown, and characteristics and features unique to PICS are documented. Although it is well known that PICS has been applied to photovoltaics and photocatalysis, here light is shed on other applications that take better advantage of PICS, such as chemical sensing and biosensing, various photochromisms, photoswitchable functionalities and nanoscale photofabrication.

## Abstract 2

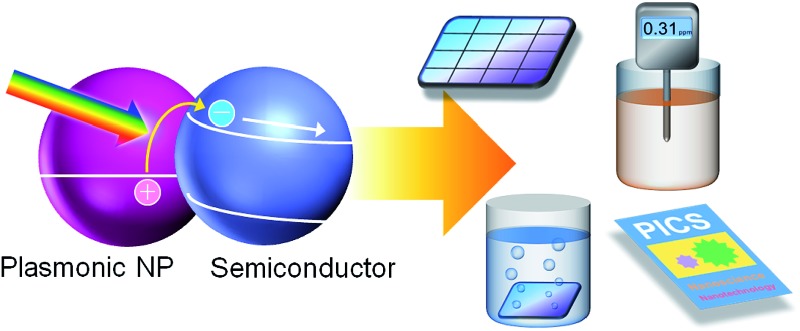
Electrons transfer from plasmonic nanoparticles to semiconductors by exploiting the energy of light, and this effect is applied to photovoltaics, photocatalysis, sensing, photochromisms, photoswitchable functionalities and nanofabrications.

## 1. Introduction to plasmon-induced charge separation

### 1.1. Plasmon-induced charge separation (PICS)

**Fig. 1:**
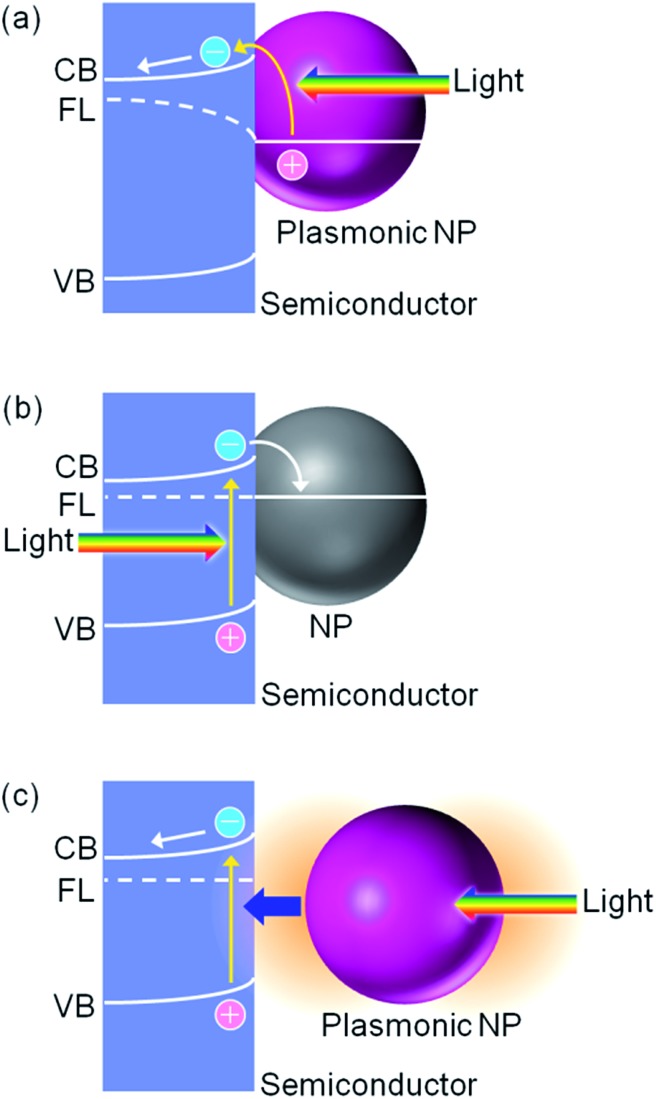
Mechanisms of (a) the plasmon-induced charge separation (PICS), (b) the co-catalysis effect and (c) the plasmonic nanoantenna effect.

If we would like to use the energy of light, we have to capture photons. To do this, we generally use dye molecules or semiconductors. Metals are not used because they reflect light, but metals of nanoscale dimensions trap photons efficiently, on the basis of localized surface plasmon resonance (LSPR). LSPR is resonance between oscillation of free electrons in the metal and electric field oscillation of the incident light.^1,2^ A photon is thus converted to a plasmon by nanoscale metal. A plasmon has a certain lifetime,^3^ and it finally decays *via* (i) nonradiative transition,^3^ (ii) radiative transition,^3^ (iii) transfer of energy to an excitable matter through the optical near field^4^ or resonance energy transfer^5^ and (iv) uphill electron transfer to a semiconductor (or other chemical species) in direct contact.^6^ The nonradiative and radiative transitions (i and ii, respectively) are observed as light absorption and scattering, respectively. Energy transfer (in a broad sense, iii) is often used for efficient excitation of dyes and semiconductors in the nanoantenna effect or the plasmonic enhancement effect. The uphill electron transfer process (iv), *i.e.* plasmon-induced charge separation (PICS, Fig. 1a), is used for direct conversion of light energy to electrical or electrochemical energy. In this perspective we focus on PICS, which has attracted growing attention over the last decade because it can be applied to photovoltaics, photocatalysis, sensing, actuation, data storage, photoswitching of functionalities and many other applications. Here we discuss characteristics of PICS and its chemistry, as well as the applications that take full advantage of PICS.

### 1.2. PICS and related phenomena

**Fig. 2:**
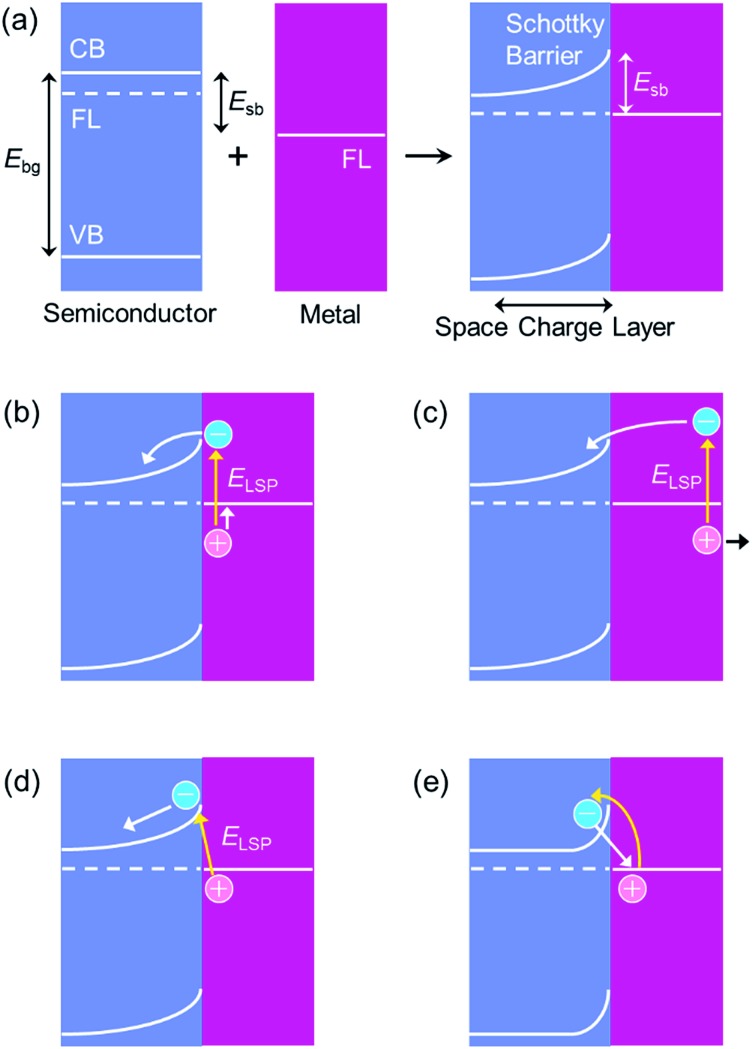
Schematic band diagrams for (a) Schottky junction, (b) PICS based on the interfacial photoelectric effect, (c) PICS based on the distal photoelectric effect, (d) PICS based on the photoinduced interfacial electron transition and (e) back electron transfer at a junction with a thin space charge layer.

We reported PICS for the first time more than 10 years ago.^6^ In general, PICS occurs at the interface between a plasmonic metal nanoparticle (NP) and a semiconductor, most typically TiO_2_.^6^ The semiconductor has a valence band (VB), which is filled with electrons, and a conduction band (CB), which does not usually have electrons (Fig. 2a). There is a certain energy gap between these bands, which is the bandgap energy *E*_bg_ (eV). When TiO_2_ is in contact with Au, a Schottky junction is formed (Fig. 2a) because the Fermi level (FL) of TiO_2_, which lies slightly below the bottom of CB, is higher than that of Au, below which bands of Au are filled with electrons. PICS involves energetically uphill electron transfer from the NP to the semiconductor CB. The electron transfer is explained in terms of external photoelectric effect or hot electron injection,^7–9^ including interfacial (Fig. 2b) and volume (Fig. 2c) mechanisms,^10^ or photoinduced interfacial electron transition (Fig. 2d).^11,12^ In the photoelectric effect, energetic and ballistic electrons generated inside the plasmonic NP are injected into the semiconductor, often after losing some energy. In the case of the interfacial electron transition, electrons in the metal are directly excited to a level in the semiconductor, typically from the Fermi level of the metal to the semiconductor CB. The pair of electric charges thus separated, namely a negative one in the semiconductor CB and a positive one in the metal NP, have an electrochemical energy, which can further be converted to electrical or chemical energy.

**Fig. 3:**
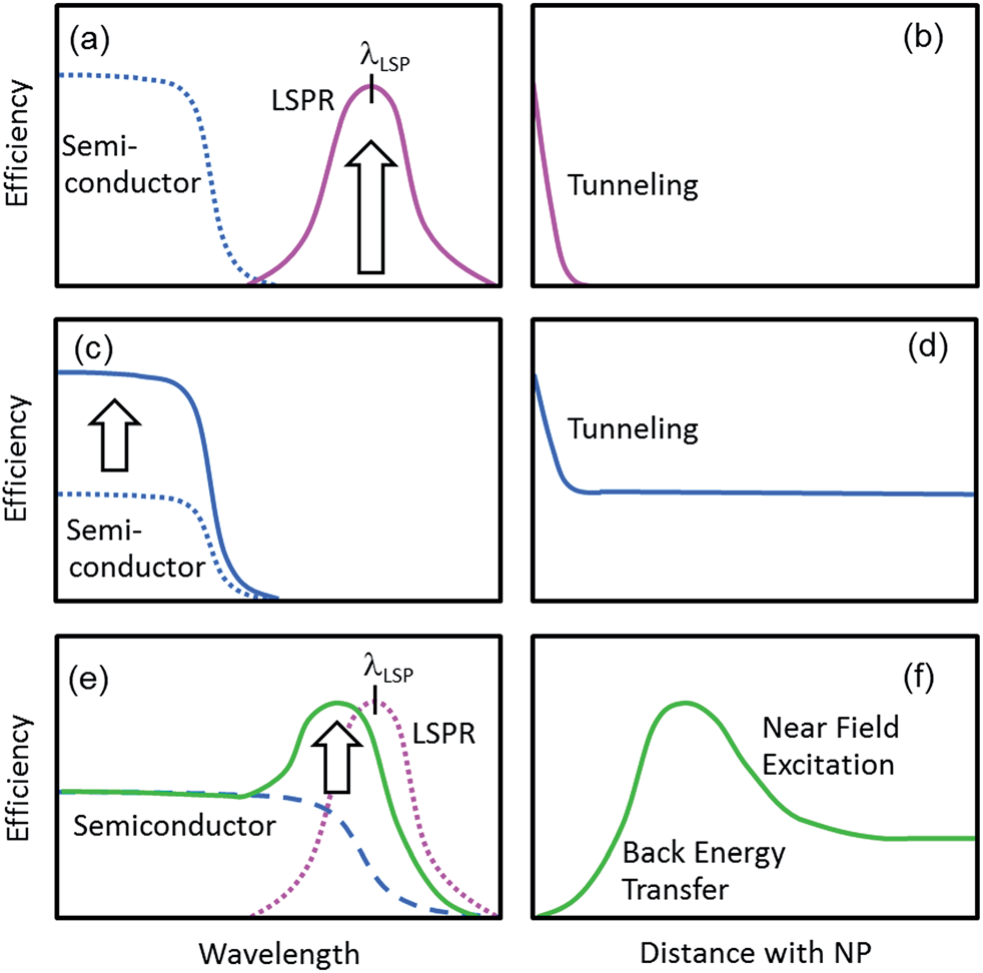
Schematic graphs for (a, c and e) the wavelength dependence and (b, d and f) the NP–semiconductor distance dependence of efficiency for (a and b) PICS, (c and d) the co-catalyst effect and (e and f) the nanoantenna effect.

Although the electron transfer mechanism is now accepted widely, PICS has been confused occasionally with some other phenomena including the co-catalysis effect of semiconductor photocatalysis (Fig. 1b)^13^ and the plasmonic nanoantenna effect (or plasmonic enhancement) (Fig. 1c).^4^ To distinguish PICS from those effects, it is important to assess the charge separation efficiency at different light wavelengths and that at different distances between the plasmonic NP and semiconductor used (Fig. 3).

In the case of PICS, photoinduced currents flow, or reactions proceed at plasmon resonance wavelengths (Fig. 3a). The energy of photons for plasmon resonance *E*_LSP_ (eV) (note that *E*_LSP_ = 1240/*λ*_LSP_, where *λ*_LSP_ in nm is the LSPR wavelength), which is used for the uphill electron transfer, should be larger than the energy gap between the semiconductor CB and the Fermi level of the NP, namely Schottky barrier height *E*_sb_ (eV) (*i.e.*, *E*_LSP_ > *E*_sb_). In general, a wide-bandgap semiconductor (*e.g.*, TiO_2_) with *E*_bg_ larger than *E*_LSP_ (*i.e.*, *E*_bg_ > *E*_LSP_) is used, so that the responsive wavelength can be controlled by changing the resonance wavelength of the plasmonic NPs, which depends on the metal species and particle morphology.^1,2^ In addition, PICS takes place only when the NPs are in direct contact with the semiconductor or the distance between them is so short that electron tunnelling occurs (Fig. 3b).

### 1.3. Difference from the co-catalysis effect

Typical semiconductor photocatalysis is based on the Honda–Fujishima effect.^14^ Electrons are excited from the VB to CB of the semiconductor, and the excited electrons in the CB and positive holes generated in the VB accordingly drive reduction and oxidation reactions, respectively. If a co-catalyst like Pt NPs or other noble metal is deposited on the semiconductor, the excited electrons accumulated in the CB spill over into the co-catalyst, and reduction reactions proceed more rapidly at the co-catalyst surface than those at the semiconductor surface, because of reduced activation overvoltage. Co-catalysts do not need to absorb light; they may be plasmonic or non-plasmonic. The photocatalytic reactions occur at wavelengths shorter than the absorption edge wavelength of the semiconductor used. In the co-catalysis effect, the reaction rates are enhanced in the whole absorption range of the semiconductor (Fig. 3c). This effect is observed when the NPs are in direct contact with, or electrically connected to, the semiconductor photocatalyst (Fig. 3d).

### 1.4. Difference from the nanoantenna effect

The nanoantenna effect is described in Section 1.1 as process (iii). This effect is observed in a wavelength range in which both of the plasmonic NPs and the semiconductor absorb light (Fig. 3e). Even in this case, however, the possibility of PICS is not ruled out. Therefore, dependence of the reaction rates on the NP–semiconductor distance should be examined (Fig. 3f). Whereas PICS and the co-catalysis effect take place only in the case of direct contact or a short NP–semiconductor distance that allows electron tunnelling (∼2 nm or less) (Fig. 3b and d), the optimum distance for the nanoantenna effect is typically ∼10 nm.^15^ As the NP–semiconductor distance decreases, the intensity of the optical near field to which the semiconductor is exposed increases, while the possibility of back energy transfer from the semiconductor to the metal NP increases.

Unfortunately, there has been confusion of the above-mentioned effects including PICS in some of studies, even if those are reported in high-impact journals. For instance, if a Au–TiO_2_ system is irradiated with a xenon lamp or a solar simulator without a UV filter, TiO_2_ is excited and Au NPs enhance the photoresponse of TiO_2_ on the basis of the conventional co-catalysis effect for photocatalysis or Schottky barrier rectification for photovoltaics. For another Au–TiO_2_ system, even if its photoresponse is explained in terms of the nanoantenna effect in the paper, it is difficult to rule out the possibility of PICS in the case where Au is in direct contact with TiO_2_.^16,17^ In addition, if there really is a possibility of the nanoantenna effect, the possibility of the co-catalysis effect cannot be ruled out without carefully obtained action spectra.

### 1.5. Difference from the plasmonic heating effect

Plasmonic NPs efficiently collect light energy, and convert it to heat if the processes (ii)–(iv) in Section 1.1 are not so efficient. Therefore, PICS is sometimes confused with a thermal effect. It is reported that plasmonic heating by laser shots results in morphology changes or fragmentation of NPs.^18^ However, the laser beam intensity is far stronger than that of sunlight (∼0.1 W cm^–2^) or other practical light sources like lamps and LEDs by at least several orders of magnitude. Actually, it is reported that the temperature increase is theoretically expected to be less than 5 and 0.05 K for 100 and 10 nm spherical Au NPs, respectively, in water at 1 kW cm^–2^.^19^ Even though the temperature increase is larger for dense particle arrays or ensembles, the temperature increase is still lower than 10 K for a hexagonal array of 11.4 nm Au NPs on a glass plate (∼160 particles per μm^2^) in water at ∼4 kW cm^–2^.^20^ Therefore, it is reasonable to neglect a contribution from the thermal effect in most cases, except for highly dense NP ensembles irradiated with strong light.

## 2. Details of PICS

### 2.1. How to select materials for PICS

**Fig. 4:**
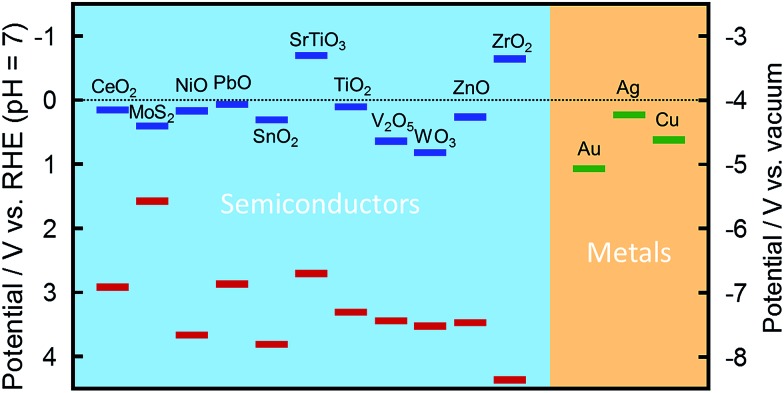
Positions of the conduction band (blue) and the valence band (red) of selected semiconductors^21–23^ and the Fermi level of selected metals.^24^

PICS occurs basically at the interface between a plasmonic NP and a semiconductor at which a Schottky junction is formed. A Schottky junction is formed if the Fermi level of the metal is deeper (more positive in potential) than the bottom of the semiconductor CB, in the case of an n-type semiconductor (Fig. 2a). In the case of a p-type semiconductor, the top of the semiconductor VB must be deeper than the Fermi level of the metal. Those levels for selected metals and semiconductors are shown in Fig. 4.^21–24^ The combination of Au NP and TiO_2_ (ref. 6) has been used in most of the PICS studies so far because of their stability and appropriate energy relationships: *E*_bg_ (∼3.2 eV) > *E*_LSP_ (1–2.4 eV) > *E*_sb_ (∼1 eV). It must be noted that the real value of the barrier height often deviates from the simple theoretical estimation.^25^

#### a. Metals

Metal NPs used for PICS must be plasmonic and stable. In addition, they must have an appropriate Fermi level and LSPR wavelength as described above. Besides Au NPs, Ag,^26^ Cu^27^ and their alloys^28,29^ and core–shell NPs are used for PICS. Although Au NPs, which are most widely used for PICS, are highly plasmonic and stable, their surface could be hydroxylated to Au(OH)_3_ under basic conditions.^30^ Au(OH)_3_ as well as Au_2_O_3_ could play an important role in some photoelectrochemical and photocatalytic reactions.^31^ In addition, in the presence of ligands like halide anion X^–^, the oxidation potential of Au is negatively shifted and Au NPs could be oxidized to [AuX_2_]^–^ or [AuX_4_]^–^ by PICS.^32^ The oxidation can be suppressed by supplying electrons from appropriate electron donors.

Less stable Ag NPs are oxidized by PICS to Ag^+^ even in the absence of ligands.^33–35^ This reaction leads to various applications unique to PICS, as described in Section 3.4. PICS oxidation of Ag is decelerated by appropriate donors or by protecting the NPs with a self-assembled monolayer (SAM) of an alkanethiol or fluoroalkanethiol.^36^ Au–Ag alloy NPs are more stable than pure Ag NPs.^29^ Ag NPs can also be used stably by coating them with a semiconductor (*e.g.*, ITO/Ag NP/TiO_2_, instead of conventional ITO/TiO_2_/Ag NP)^37^ or by incorporating into solid state devices (Section 3.1).^38,39^ Cu is a less noble metal, so Cu NPs are oxidized and lose LSPR even in the dark. Cu NPs protected by a thin polymer layer^27^ or Au–Cu alloy NPs^40^ are used for PICS.

Single crystalline metal NPs with a well-defined size and shape are chemically synthesized as colloids and are adsorbed onto the semiconductor surface.^26,41^ In this case, NPs are covered with ligands, which help the NPs to be suspended in a liquid. Since the ligand often retards or blocks the electron transfer at the interface, it may be removed for instance by annealing or UV irradiation. NPs with well-defined morphology can also be prepared by lithographic methods.^42^ Those NPs prepared by evaporation may be polycrystalline. A thin adhesive layer such as metallic Ti may be inserted between the metal and semiconductor, so that it could affect the electron transfer. More convenient but less controllable methods include photoelectrochemical,^6,33,43^ electrochemical^34,44^ and chemical reduction of precursor metal ions at solid surfaces. These methods give NPs of various sizes at the same time, probably including very small ones, which might give a different charge separation pathway as described in Section 2.5. The number of the small NPs could be decreased by annealing.^37^

Particle size dependence of the PICS efficiency is governed by some different factors.^41,45^ Large nanoparticles are advantageous in terms of having a well-established space charge layer when in contact with a semiconductor, resulting in efficient charge separation. Small NPs are preferential from several view points: (i) a large specific surface area, (ii) good contact with a semiconductor that has nanoscale roughness, (iii) a Fermi level that is readily shifted by electron ejection, (iv) possible high efficiency of hot electron generation and (v) a short path length for ballistic electrons in the case of the distal photoelectric effect shown in Fig. 2c.

A morphological anisotropy gives rise to splitting of an LSPR mode. A plasmonic nanorod has transverse and longitudinal modes, in which electrons oscillate along the short and long axes of the nanorod, respectively. Those modes are therefore excited in response to light polarized along the short and long axes, respectively. The resonant wavelength of the longitudinal mode red shifts as the aspect ratio increases, and easily shifts into the near infrared region.^1^ A nanoplate also has two resonance modes, namely in-plane and out-of-plane modes.^2^ When a NP is in contact with a solid substrate that has a higher refractive index than the surrounding gas or liquid, its LSPR splits into an oscillation localized at the NP–solid interface and one at another part of the NP.^46^ If the condition *E*_LSP_ > *E*_sb_ is satisfied, those modes may cause PICS. Even so, the PICS efficiency could be low when the *E*_LSP_ is close to *E*_sb_.^47^ In addition to dipolar resonances, multipolar resonances (higher order resonances) can also give rise to PICS.^48^

#### b. Semiconductors

Semiconductors used for PICS must also be stable, and their conduction and valence band levels must be appropriate. In most cases the *E*_bg_ value is larger than *E*_LSP_, so that the semiconductor itself is not excited and PICS can be separated from the nanoantenna effect. In some cases, however, semiconductors with *E*_bg_ smaller than *E*_LSP_ is used.

As a semiconductor, ZnO^34^ and CeO_2_ (ref. 49) have also been used for PICS besides TiO_2_. In the case of NPs resonant in the near infrared region, Si,^50^ GaAs^51^ and MoS_2_ (ref. 52) are used as semiconductors. The use of graphene is also reported.^53^

The bottom of the semiconductor CB must be “moderate”. If it is too high (*i.e.*, too negative in potential), electrons cannot be injected from NPs to the CB by visible light. If it is too low (*i.e.*, too positive in potential), a Schottky barrier is not developed sufficiently. Electronic conductivity of the semiconductor should also be “moderate”, so that the space charge layer, in which the bands bend, has a sufficient thickness for electrons injected from the NPs to be transported to the semiconductor bulk along the bent CB (Fig. 2a). In the case of ITO, which has metallic conductivity, PICS is not observed or the efficiency is very low,^34,54^ likely because the space charge layer is too thin to separate the charges, resulting in back electron transfer from ITO to the metal by electron tunnelling through the barrier (Fig. 2e).

### 2.2. What happens, and where

**Fig. 5:**
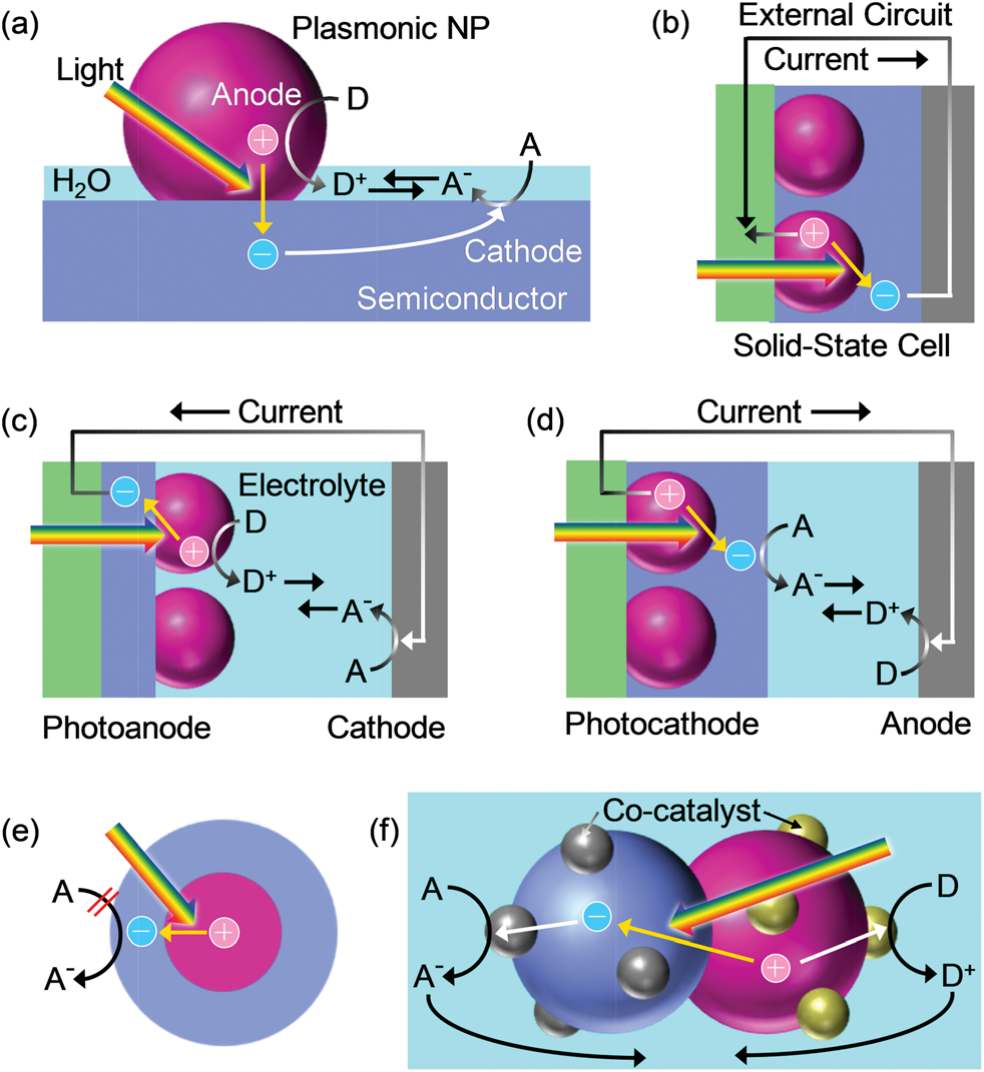
Schematic illustrations for charge transport in PICS systems. (a) A general photocatalytic system, (b) a solid-state cell, (c) a cell with a photoanode, (d) a cell with a photocathode, (e) a core–shell NP and (f) a photocatalyst NP with anodic and cathodic co-catalysts.

PICS always drives reduction and oxidation at the same time, as with other photoelectrochemical and electrochemical reactions, otherwise electrons or holes are left in the photocatalyst and the reactions cannot be continued. In the case of a photoelectrochemical process based on PICS, (i) electrons transfer from the metal NP to semiconductor CB, (ii) oxidation (*i.e.*, anodic reaction) takes place at the metal NP surface, (iii) reduction (*i.e.*, cathodic reaction) takes place at the semiconductor surface or at metals connected to the semiconductor and (iv) ions are transported between the anodic and cathodic sites for charge compensation (Fig. 5a). In the case of a solid-state photovoltaic cell, chemical reactions do not occur and electrons are transported from the anode to the cathode *via* an external circuit (Fig. 5b). In either case, an electrochemical or electrical circuit must be established.

For the PICS system with Au or Ag NPs and TiO_2_, the anodic sites have been found on the NPs through oxidation of themselves as described in Section 2.1. Oxidative polymerization of pyrrole also proceeds at Au NPs.^55,56^ The cathodic sites have been located at the TiO_2_ surface by taking advantage of reduction of Ag^+^ to Ag NPs.^57,58^ Although cathodic sites have been found around the NPs,^59^ those can be apart from the NPs by >1 μm.^57^

**Fig. 6:**
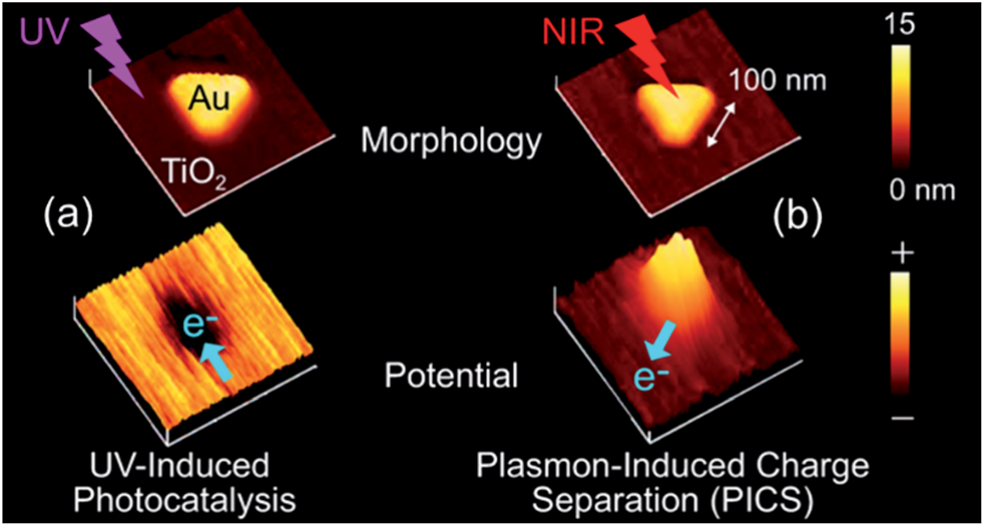
Results of KFM analysis for the Au nanoplate–TiO_2_ system. (Left) The co-catalysis effect and (right) PICS. Reproduced with permission from ref. 60 © John Wiley and Sons.

These anode and cathode locations are reasonable because the potential distribution measurements by means of Kelvin probe force microscopy (KFM) prove that the surface potential at the Au NP in resonance with light is more positive than that at the TiO_2_ surface (Fig. 6b).^60^ It is noteworthy that the opposite potential distribution is observed for the co-catalysis effect under UV light (Fig. 6a). In addition, an ITO electrode coated with a TiO_2_ film and further modified with Au or Ag NPs (*i.e.*, ITO/TiO_2_/NP, Fig. 5c) exhibits negative photopotential shifts and anodic photocurrents,^6,26^ whereas an ITO electrode modified with Au or Ag NPs and further coated with TiO_2_ (*i.e.*, ITO/NP/TiO_2_, Fig. 5d) shows opposite responses, namely positive photopotential shifts and cathodic photocurrents.^37,44,61^ These results are also in line with the locations of the anodic and cathodic reaction sites shown in Fig. 5a.

Since the charge separation occurs at the metal–semiconductor interface, PICS occurs even for the metal@semiconductor core shell NPs.^62^ Actually, electron injection from the metal core to the CB of the semiconductor surrounding the NPs results in negative potential shifts and a conductivity increase in the semiconductor.^63,64^ However, positive charges left in the metal core cannot be used to drive oxidation reactions (Fig. 5e) unless the semiconductor shell is thin enough to allow electron tunneling or the shell has pinholes through which electron donors outside approach the metal core. The separated charges are therefore recombined when the light is turned off.

**Fig. 7:**
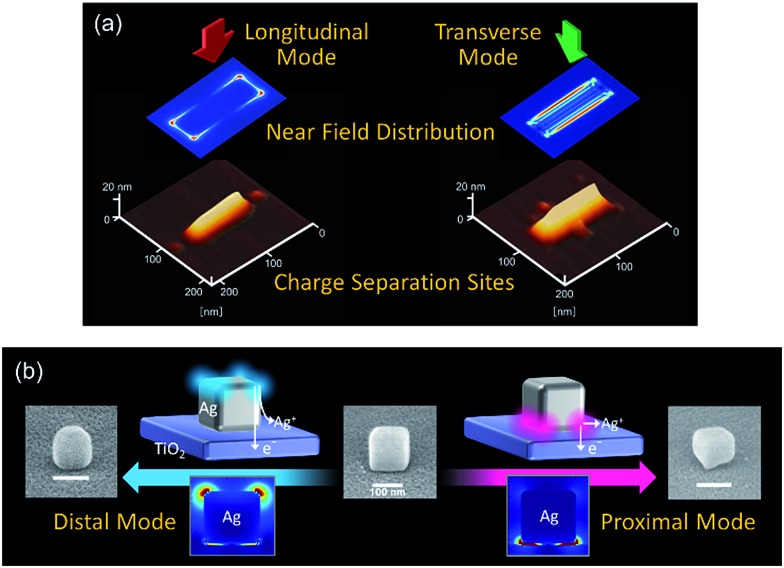
Results of reaction site analysis for PICS with (a) Ag nanorods on TiO_2_ (ref. 58) and (b) Ag nanocubes on TiO_2_.^67^ (b) Reproduced with permission from ref. 67 © 2016 American Chemical Society.

The electron transfer and accompanying reactions are suggested to occur preferentially at sites where the optical near field is localized (Fig. 7). These were confirmed by oxidation of Ag NPs^58,65^ and suggested by oxidation of Au NPs^66^ and organic species.^56^ In particular, studies using Ag and Au nanospheres and Ag nanocubes elucidated that oxidation reactions are induced or promoted by the near field (Fig. 7b). This can be explained in terms of ejection of hot holes or holes trapped at the Ag surface as Ag^+^ to the solution, accompanied by the distal ejection of energetic electrons to TiO_2_ (Fig. 2c).^67^

In addition, hot carriers are expected to be generated frequently if the electric field is localized strongly.^68^ Therefore it is anticipated that Au nanostars give higher activity in PICS-based photocatalysis than nanorods and nanospheres.^68^

The interfacial electron transition occurs only at the interface. In the case of the external photoelectric effect, it can occur not only at the interface. It can occur in the range within the mean free path length *d*_mfp_ of the energetic carrier. In the case of the Au–TiO_2_ system, *d*_mfp_ of free electrons in Au with energy that is sufficient to overcome the Schottky barrier with *E*_sb_ ∼ 1 eV is ∼42 nm.^69^ In the case of Ag–TiO_2_, *d*_mfp_ is 56–58 nm.^69^ Since the energy of resonant photons can be up to ∼2.4 or ∼3.1 eV in the case of Au and Ag NPs, hot electrons with an energy of this much should travel for distances longer than the lengths described above losing some energy.^10^

### 2.3. Reactions driven by PICS

The anodic and cathodic potentials define which reactions take place: the more positive (negative) the anodic (cathodic) potential, the greater the variety of the oxidized (reduced) chemical species. The cathodic potential can be measured directly by electrochemical means. When the ITO/TiO_2_/Au NP electrode is irradiated with light at the resonance wavelength, electrons are injected from Au NPs and accumulate in the TiO_2_ CB or trap sites slightly below it, resulting in a negative potential shift, if the light intensity is sufficiently high and/or the solution does not contain a sufficient amount of electron acceptors. The negative limit of the potential is the CB edge potential at the TiO_2_ surface, *i.e.*, the flat-band potential *Φ*_fb_ (V), which depends on solution compositions such as pH. Accumulation of electrons in TiO_2_ means that electrons could be depleted in Au NPs in the absence of appropriate electron donors of a sufficient amount. This is an “open-circuit” situation in electrochemical terms, in which the Fermi level is different between the metal and semiconductor (Fig. 1a). Note that the Fermi level difference is absent or negligible if the system is perfectly short-circuited or right after starting irradiation (*e.g.*, Fig. 2). The positive limit of the “open-circuit” potential of the Au NP is more positive than *Φ*_fb_ by *E*_LSP_.^30^ In other words, the maximum potential difference between Au and TiO_2_ is *E*_LSP_. The oxidation and reduction abilities of PICS can therefore be controlled by tuning light wavelength and intensity as well as the solution compositions.

If there are trap sites for instance at the metal NP surface, their level may define the effective anodic potential, whereas the hole would decay to the Fermi level instantaneously and shift the Fermi level positively. When the metal NP is in direct contact with a hole acceptor such as a p-type semiconductor or an adsorbed electron donor, an energetic hole could be injected directly to it, if the electron transfer is sufficiently fast.^70^

### 2.4. Uniqueness of PICS

On the basis of the discussions mentioned above and other general characteristics of LSPR, here we summarize the unique features of PICS as follows: (i) controllability of wavelength in the visible and near infrared ranges by simply changing the size and shape of plasmonic NPs. (ii) Controllability of anodic and cathodic potentials by changing the solution composition (typically pH) and irradiated light (intensity and wavelength). (iii) Controllability of response to linearly or circularly polarized light by changing morphology and orientation of anisotropic and chiral NPs (see Section 3.1). (iv) Sensitivity to refractive index (see Section 3.3). (v) Applicability to control of NP morphology and orientation beyond the diffraction limit, as well as optical properties (see Section 3.5).

### 2.5. Marginal area of PICS and beyond

PICS is studied usually by using lamps (*e.g.*, xenon lamp), but it can also be observed when the system is irradiated with pulse laser, the intensity of which is higher than that of a xenon lamp by at least several orders of magnitude. Therefore many photons are injected into a NP at the same time,^71^ and even a two photon process can occur.^72^

PICS is usually based on LSPR, but propagating surface plasmon resonance at metal film surfaces is also reported to cause PICS.^73^ In the case of a nanostructured but continuous plasmonic film,^74^ both localized and propagating plasmons may contribute to PICS. However, details are yet known because analysis of the localized and propagating modes is complicated.

**Fig. 8:**
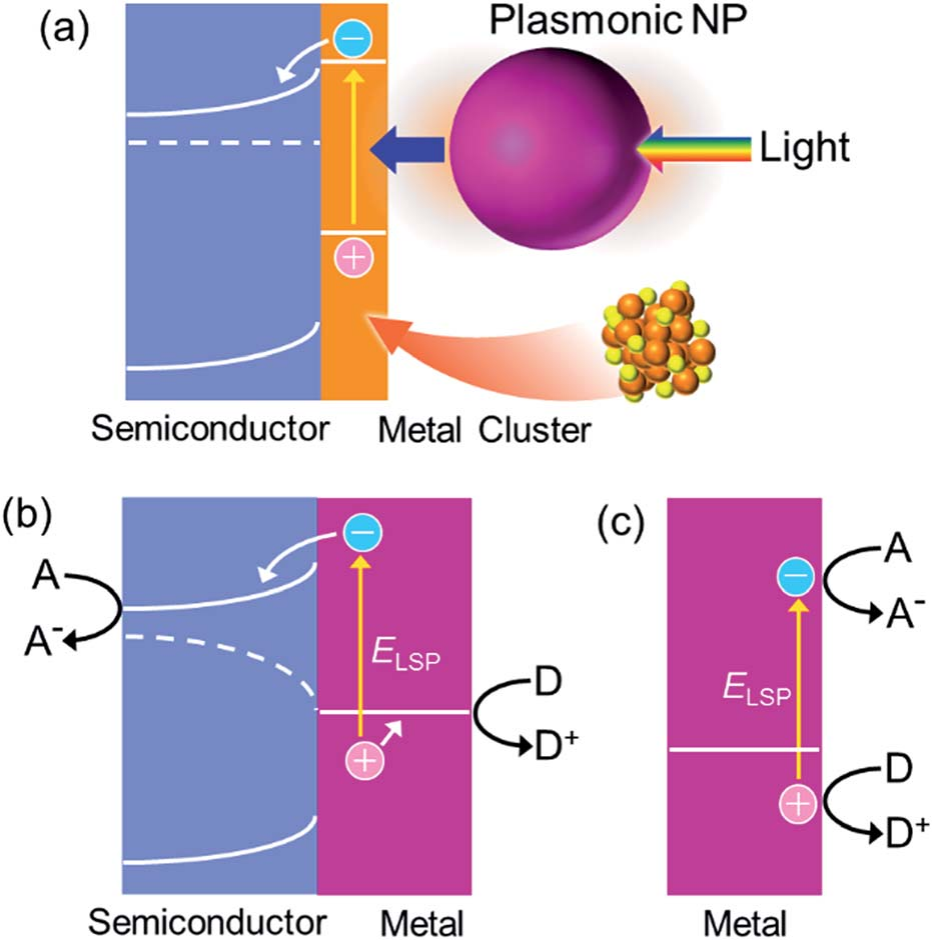
(a) Combined metal cluster–nanoantenna system. Difference in energetics between (b) PICS and (c) direct plasmonic electrochemical reactions.

When metal NPs are deposited by photoelectrochemical means,^75^ NPs with various different sizes are deposited on a semiconductor. In that case, small and large NPs could play different roles: small NPs directly inject electrons to a semiconductor, and large NPs enhance the light absorption of the small NPs by the nanoantenna effect. The small NPs could be non-plasmonic NPs, which are smaller than ∼2 nm, called metal clusters (Fig. 8a).^76^ Actually it is reported that coexistence of small Au NPs (∼2 nm) improves PICS activity of large Au NPs (∼13 nm) on TiO_2_.^77^

Recently, direct plasmonic electrochemical reactions^78^ involving hot electron (or hole) transfer^79^ or interfacial electron transition^12,80^ to external chemical species such as organic molecules without intermediation by a semiconductor have also attracted the attention of researchers in the field of catalysis in particular. Photoinduced growth of plasmonic metal NPs may also be a similar effect.^81^ As Fig. 8c shows, higher voltage could be used in the system without a semiconductor because electrons and holes are directly transferred to electron accepting and donating species, respectively. However, the use of a semiconductor is advantageous for efficient charge rectification because the Schottky junction retains the positive and negative charges separated for a longer time in exchange for the voltage (Fig. 8b). Regardless of such differences from PICS with a semiconductor, discussion of the system without a semiconductor would also help understanding of PICS with semiconductors.

## 3. Applications of PICS

### 3.1. Photovoltaic and photoelectric devices

**Fig. 9:**
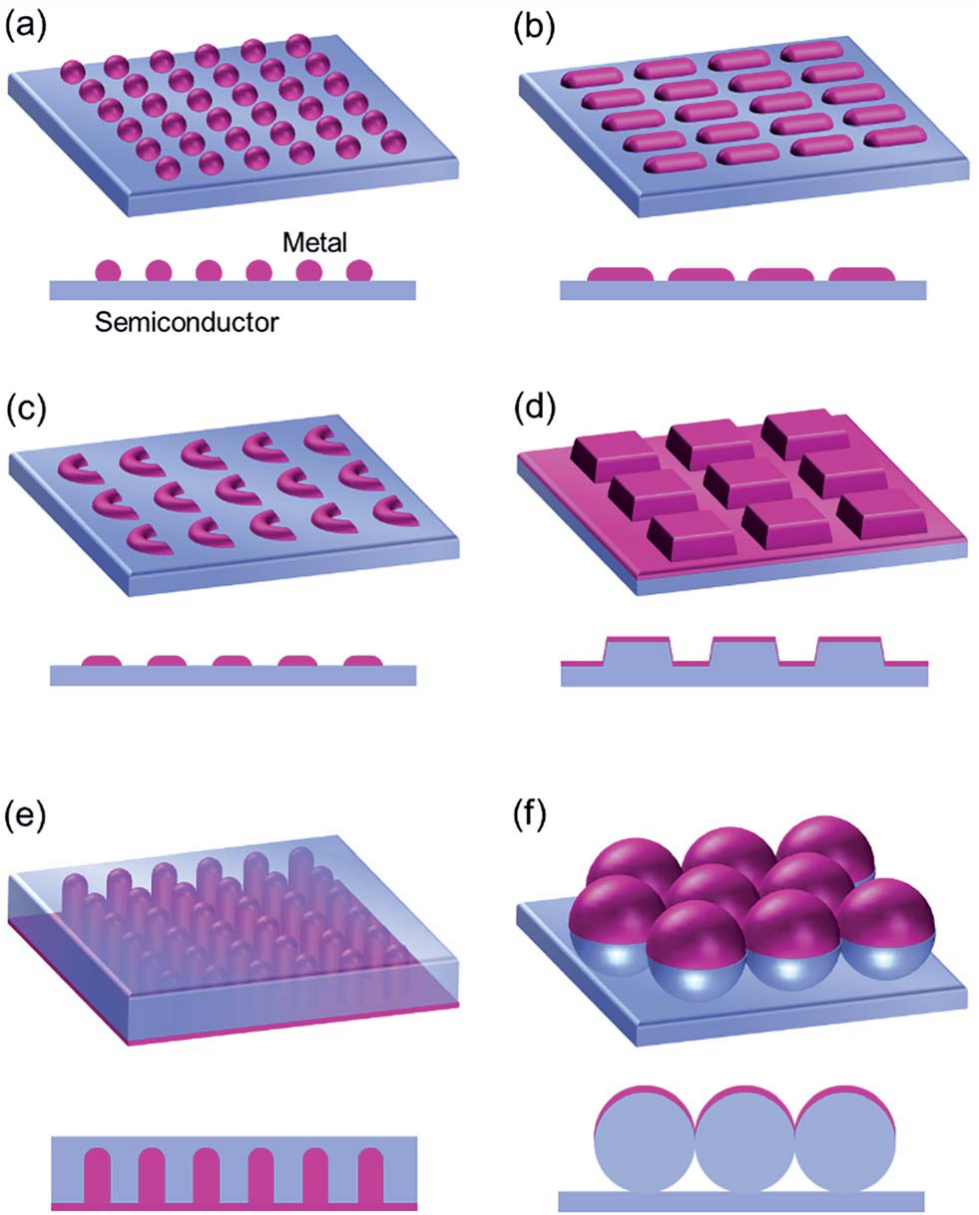
Metal–semiconductor nanostructure interfaces. (a) Metal nanosphere array, (b) metal nanorod array, (c) chiral metal nanocrescent array, (d) perfect absorber, (e) upright metal nanorod array and (f) metal halfshell array.

Photoinduced uphill electron transfer like PICS can be applied to photovoltaic cells. Initially wet-type cells similar to dye-sensitized solar cells were reported,^6^ followed by development of solid-state cells.^82^ The latter include cells with an n-type/NP/p-type structure^39,82^ and those with a simpler n-type/NP structure.^38,50,83^ As the p-type semiconductor or hole transport material, CuI, CuSCN, poly(*N*-vinylcarbazole),^82^ Spiro-OMeTAD^39^ and NiO^84^ have been used. A cell with a polyethylene oxide solid electrolyte was also reported.^85^ Efficiency of the photovoltaic cell is relatively low, so photodetector applications like photodiode-type devices^86^ and photoconductive cells^63,87^ may be promising, in particular for detection of near infrared light. It is relatively easy to prepare devices sensitive to near infrared light using anisotropic NPs (Fig. 9b–f) by taking advantage of PICS.^38,42,50^ PICS in a deeper infrared range has been explored by replacing Au NPs of a Au–TiO_2_ system (Fig. 9a)^6^ with Au nanorods (Fig. 9b)^42^ and TiO_2_ with Si.^50^ A metamaterial called a “perfect absorber” is also incorporated into a Au–Si near infrared photodetector (Fig. 9d).^88^ A continuous plasmonic nanostructure serves both as a light absorber and an electrode (Fig. 9d–f).^74,86,88^ Devices relatively selective to linearly^42^ or circularly^89^ polarized light have also been reported (Fig. 9b and c, respectively).

### 3.2. Photocatalysis

Positive and negative charges provided by PICS can be used for driving oxidation and reduction reactions, respectively. First PICS was applied to oxidation of alcohols^6,43^ and aldehydes,^6^ as well as mineralization of organic molecules to CO_2_.^49^ Then, oxidation of water to O_2_ (ref. 90–92) and reduction of water to H_2_ (ref. 90 and 93) were reported, followed by a report on photoinduced water splitting.^94^ Reduction of CO_2_ to formic acid was also reported.^53^ Other photocatalytic reactions have also been reported: oxidation of benzene to phenol,^95^ oxidation of thiol to disulfide,^96^ release of H_2_ from alcohols and ammonia,^77^ dechlorination of chlorobenzene,^97^ *etc.* Generation of hydroxyl radicals is also suggested.^98^

Currently, standard photocatalyst materials are n-type semiconductors like TiO_2_. If noble metal NPs are deposited on it, the NPs may work as co-catalysts for reduction reactions as described in Section 1.3. However, in the case of PICS, plasmonic noble metals can serve as co-catalysts for oxidation reactions. A dual co-catalyst system, in which Au NPs work as light absorber and anodic co-catalysts and Pt NPs work as cathodic co-catalysts has also been reported.^34^ Plasmonic metal NPs may further be modified with anodic co-catalysts (Fig. 5f).^94^ Other reactions reported before 2014 are well documented in a review article.^99^

Most of those photocatalytic reactions based on PICS^99,100^ can also be driven by conventional photocatalysts. Since the anodic potential of PICS can be controlled as described in Section 2.3,^30^ it would be possible to develop photocatalysts with a certain reaction selectivity, which will be unique to PICS.

### 3.3. Chemical sensors and biosensors

**Fig. 10:**
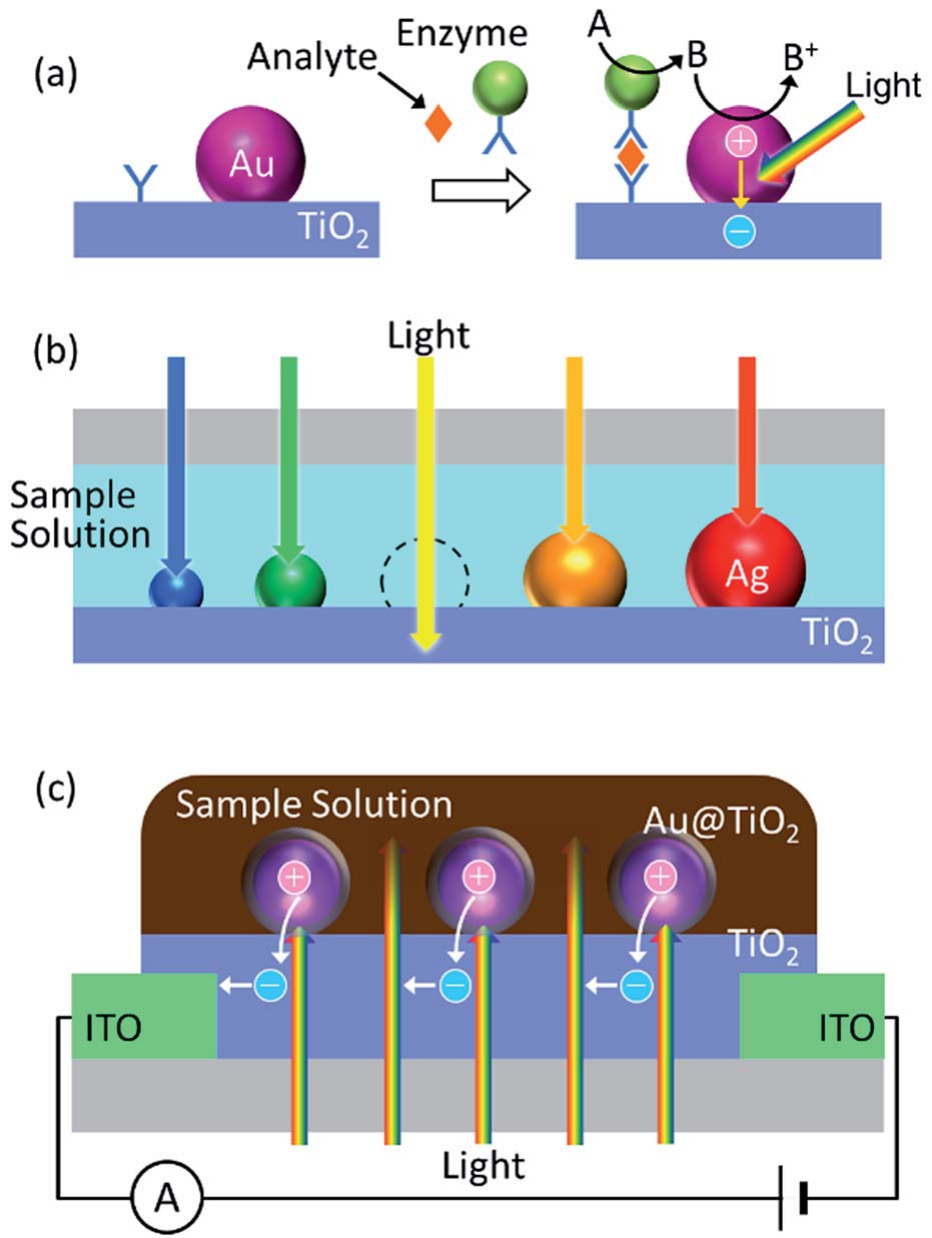
Chemical sensors and biosensors based on PICS. (a) PICS is used for detection of a marker.^101,102^ (b) Dip-based sensor for refractive index sensing.^36^ (c) Conductometric sensor for refractive index sensing.^64^

PICS has also been applied to chemical sensing and biosensing. First PICS was used for photoelectrochemical detection of redox markers generated in biosensing (Fig. 10a).^101,102^ It is also reported that PICS-based photocurrents from antibody-modified Au NPs on TiO_2_ are increased by selective binding of the antibodies to the corresponding antigens.^103^

Plasmonic NPs are also used for sensing based on refractive index changes because the LSPR wavelength is increased in general as the local refractive index around the NP increases.^104^ If the NP surface is modified with receptors (*e.g.*, antibodies), the absorption peak of the NP is redshifted as the receptors combine with their guest species (*e.g.*, antigens). Although the LSPR sensors are less sensitive than so-called surface plasmon resonance (SPR) sensors based on propagating SPR, the LSPR sensors are advantageous in cost and compactness. PICS is applied to LSPR sensors for measurements at an arbitrary wavelength at which the sample solution is more transparent and the sensor is more sensitive. Polydisperse Ag NPs are deposited on TiO_2_, and are irradiated with relatively strong monochromatic light, so that the resonant NPs are selectively oxidized to Ag^+^, and an absorption dip is formed at the excitation wavelength (Fig. 10b). Since the absorption dip redshifts with increasing local refractive index, as does the absorption peak,^36^ it allows LSPR sensing.

PICS also allows the refractive index-based LSPR sensors to output electrical signals directly. Potentiometric and conductometric LSPR sensors have been reported, and those sensors give responses due to PICS even in the absence of supporting electrolyte, even if the sample solution is coloured or turbid (Fig. 10c).^64^

### 3.4. Storage of information and switching

**Fig. 11:**
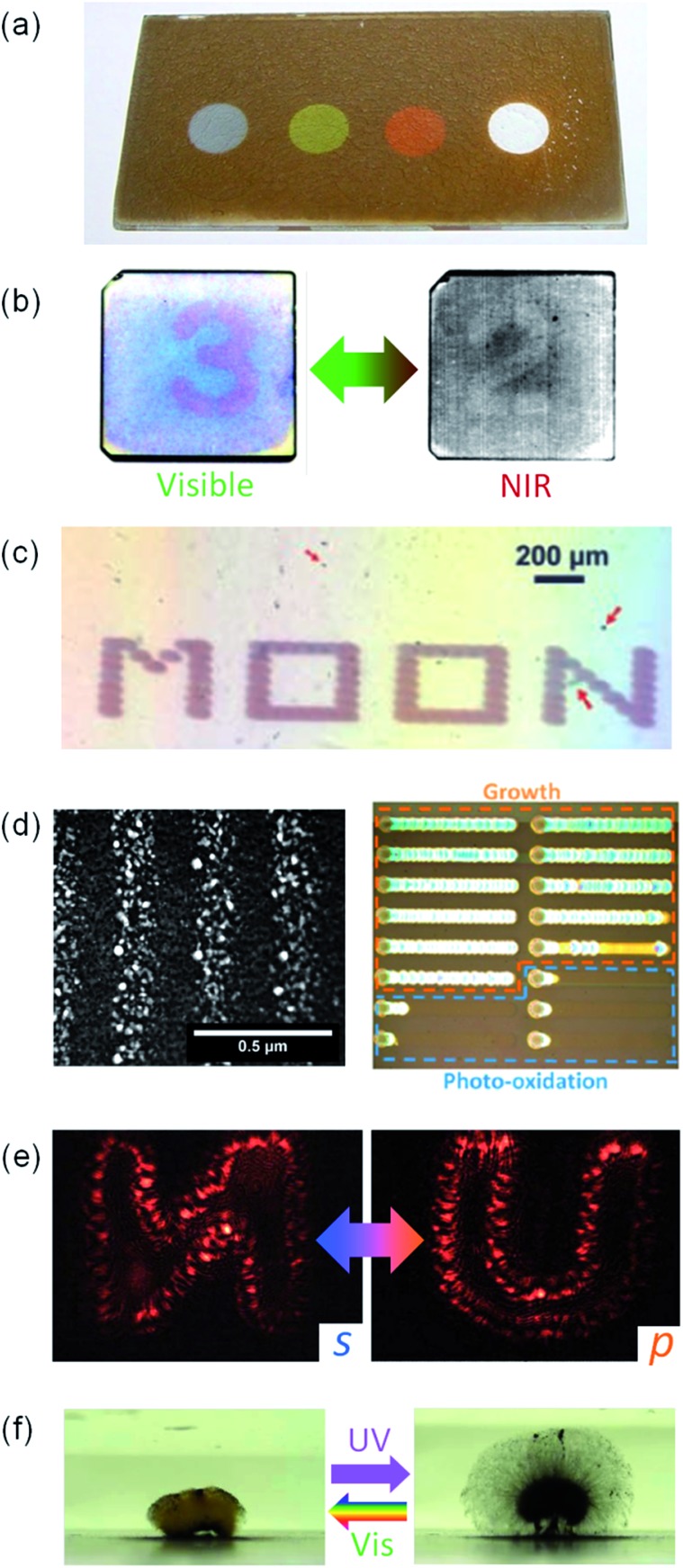
Applications of Ag NP photooxidation based on PICS. (a) Multicolour photochromism, (b) infrared photochromism, (c) fast photochromism utilizing laser light, (d) photogenerated gratings, (e) holographic images and (f) photomorphing gel. Reproduced with permission from (a) ref. 106 © 2004 American Chemical Society, (b) ref. 108 © The Royal Society of Chemistry, (c) ref. 109 © John Wiley and Sons, (d) ref. 112 © the PCCP Owner Societies and ref. 111 © 2015 American Chemical Society and (f) ref. 35 © John Wiley and Sons. (e) Reproduced from ref. 114 CC BY 4.0.

If Ag NPs are used for PICS, the NPs are oxidized to Ag^+^ and their morphology is changed as a result of PICS. This can be used for photoinduced reversible colour changes, namely photochromism.^105^ This allowed the development of the first multicolour photochromism in which the colour of the material is changed to almost the same colour of the irradiated light (Fig. 11a).^33,106^ This is explained in terms of size-selective oxidation by PICS from polydisperse Ag NPs on TiO_2_.^75,107^ Images drawn by PICS are gradually bleached under room light, because all the Ag NPs are oxidized to Ag^+^. However, the sample can be initialized by irradiating with UV light, which excites TiO_2_ and reduces Ag^+^ to Ag NPs. The drawn image can be retained for a longer time by protecting it with a SAM as mentioned in Section 2.1. Au NP–TiO_2_ systems also allow drawing of multicolour images in the presence of halide ligands.^32^ In this case, the image is stable even under room light.

Multicolour photochromism has been extended to the near infrared region, so that invisible images that can be visualized by infrared camera are overlaid on visible images (Fig. 11b).^108^ This is based on aspect ratio-selective oxidation of Ag nanorods on TiO_2_. Since a Ag nanorod has two LSPR modes, transverse and longitudinal modes, which are resonant with light polarized along the short and long axes of the nanorod, respectively, polarization angle-selective drawing is possible with the Ag nanorod ensembles on TiO_2_.

PICS was also applied to fast photochromism, in which monochromatic images are drawn by 244 nm laser (150 ms, Fig. 11c) and are erased by 488 nm laser irradiation (1 min).^109^ Two-dimensional hydrophilic/hydrophobic patterning,^110^ photofabrication of gratings^111,112^ and holographic storage of data and images^113,114^ are additional applications. The patterning is based on removal of a hydrophobic thiol anchored to plasmonic NPs by PICS. The gratings consisting of aligned Ag NPs (Fig. 11d) are formed as a result of balanced oxidation and re-reduction of Ag. The holographic images are also drawn by PICS and one can see different images under s-polarized and p-polarized light (Fig. 11e).

The PICS-based photoinduced reversible process between Ag NPs and Ag^+^ can also be applied to a photomorphing hydrogel or a photoactuator, in which interaction between the polymer chains is reversibly regulated by UV light that induces conventional photocatalysis and visible light that causes PICS (Fig. 11f).^35^ Photoswitching of an antimicrobial effect of Ag^+^ (ref. 115) and phototuning of photocatalytic effects^116^ are also based on the reversible process. Most of these applications are unique to PICS.

### 3.5. Nanofabrication

The technique described in the preceding section is also used for manipulation of particle morphology. The size of the Ag NPs resonant with the incident light can be reduced by oxidation to Ag^+^ on the basis of PICS. This allows size reduction of NPs of specific size from polydispersed NPs.^75^ In the case of Ag nanorods, aspect ratio-selective oxidation is possible. Nanorod length or width is decreased by excitation of the longitudinal or transverse mode, respectively.^58,108^ If upright Ag nanoplates are deposited on TiO_2_, irradiation of linearly polarized light results in preferential toppling or removal of the nanoplates aligned parallel or perpendicularly to the polarization angle by excitation of the in-plane longitudinal or in-plane transverse mode, respectively.^117^ Since PICS-based oxidation of Ag NPs takes place preferentially at the sites where the electric field is localized, partial etching of NPs is also possible. A Ag^65^ or Au^66^ nanosphere on TiO_2_ is peeled when its full-surface mode is excited, and its bottom is etched when the interface mode is excited. In the case of a Ag nanocube on TiO_2_, its top or bottom face is bevelled by excitation of its distal or proximal mode, respectively (Fig. 7b).^67^ Porous NPs can also be prepared from Au–Ag alloy NPs by photoinduced dealloying.^118^

### 3.6. Other applications

**Fig. 12:**
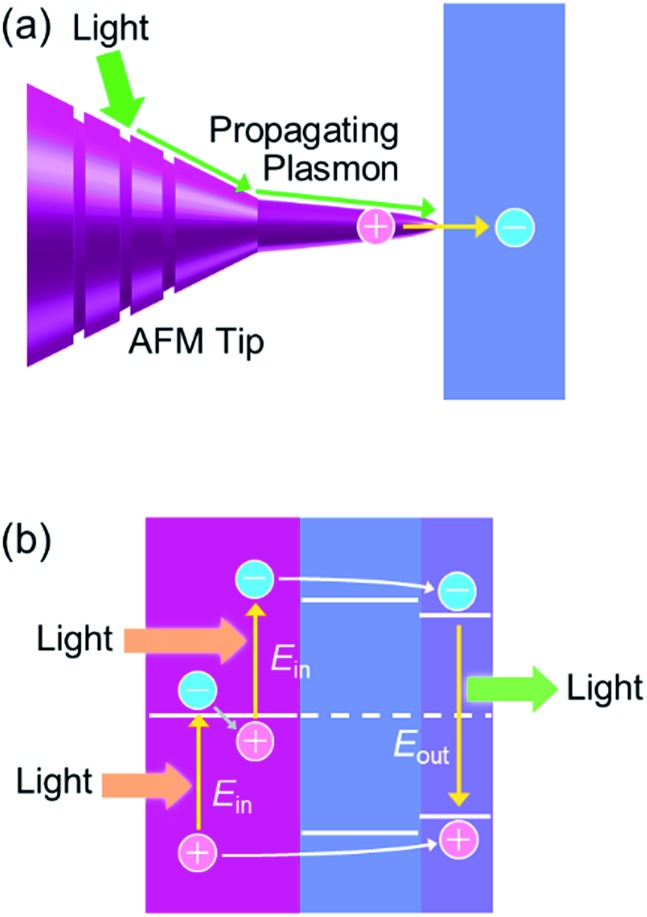
(a) Scanning-probe nanoscopy^51^ and (b) photon upconversion.^119^

Other unique applications of PICS include nanoscale two-dimensional imaging of the electron accepting ability at the semiconductor surface.^51^ A Au tip with nanoscale grooves for atomic force microscopy (AFM) is used for a GaAs substrate. Light irradiation generates surface plasmons at the grooves and they propagate to the apex, causing electron injection from the tip to the substrate (Fig. 12a).

PICS is also applied to a photon upconversion device with a structure shown in Fig. 12b.^119^ The device is irradiated with photons with energy of *E*_in_, so that energetic electrons above the Fermi level of the metal NP by about *E*_in_ are injected into the semiconductor CB. Simultaneously energetic holes below the Fermi level by about *E*_in_ are injected into the semiconductor VB. The band gap *E*_bg1_ of the semiconductor is slightly smaller than 2*E*_in_. The injected electrons and holes are trapped in a quantum well with *E*_bg2_ slightly smaller than *E*_bg1_ and are recombined with each other to emit photons with energy of *E*_out_, which is larger than *E*_in_ (2*E*_in_ > *E*_bg1_ > *E*_bg2_ ∼ *E*_out_ > *E*_in_).

## 4. Conclusions and outlook

PICS, which typically involves uphill electron transfer from plasmonic NPs to a semiconductor CB, is characterized by controllability of the active wavelength in the visible and near infrared range. Sensitivity to the local refractive index and incident light polarization are additional features. On the basis of the distinctive characteristics, PICS has expanded its range of applications to the fields of photovoltaics, photocatalysis, chemical sensing and biosensing, photochromism, photoswitchable functionalities and nanofabrication. More specifically, applications taking full advantage of PICS include photocells responsive to near infrared light and polarized light, photocatalysts with anodic and cathodic metal catalysts, sensors based on refractive index changes, multicolour photochromic materials and site-selective nanofabrication of plasmonic NPs.

The basis of the semiconductor photocatalysis was found at the end of 1960s,^14^ and a simple TiO_2_ film was put into practical use as a photocatalyst in the early 1990s. It was commercialized probably because of the simple material and the unique functionality, namely the very strong oxidation ability without wired power supply or consecutive maintenance. Applications of PICS should also be unique to itself, and the structure and mechanisms behind it should not be too complicated. The controllability and tunability of PICS would be key for more sophisticated and practical applications.

## Abbreviations

A: Electron acceptor
AFM: Atomic force microscopy
CB: Conduction band
D: Electron donor
*d*_mfp_: Mean free path length
*E*_bg_: Band gap energy
*E*_in_: Input photon energy
*E*_LSP_: Photon energy for LSPR
*E*_out_: Output photon energy
*E*_sb_: Schottky barrier energy
FL: Fermi level
NIR: Near infrared light
ITO: Indium-tin oxide
KFM: Kelvin probe force microscopy
LED: Light-emitting diode
LSPR: Localized surface plasmon resonance
NP: Nanoparticle
PICS: Plasmon-induced charge separation
RHE: Reversible hydrogen electrode
SAM: Self-assembled monolayer
SPR: Surface plasmon resonance
UV: Ultraviolet light
VB: Valence band
Vis: Visible light
*λ*_LSP_: LSPR wavelength
*Φ*_fb_: Flat band potential
